# Cross-cultural adaptation of research instruments: language, setting, time and statistical considerations

**DOI:** 10.1186/1471-2288-10-13

**Published:** 2010-02-10

**Authors:** Linn Gjersing, John RM Caplehorn, Thomas Clausen

**Affiliations:** 1 SERAF- Norwegian Centre for Addiction Research, University of Oslo, Oslo, Norway; 2 School of Public Health, University of Sydney, Sydney, NSW, Australia

## Abstract

**Background:**

Research questionnaires are not always translated appropriately before they are used in new temporal, cultural or linguistic settings. The results based on such instruments may therefore not accurately reflect what they are supposed to measure. This paper aims to illustrate the process and required steps involved in the cross-cultural adaptation of a research instrument using the adaptation process of an attitudinal instrument as an example.

**Methods:**

A questionnaire was needed for the implementation of a study in Norway 2007. There was no appropriate instruments available in Norwegian, thus an Australian-English instrument was cross-culturally adapted.

**Results:**

The adaptation process included investigation of conceptual and item equivalence. Two forward and two back-translations were synthesized and compared by an expert committee. Thereafter the instrument was pretested and adjusted accordingly. The final questionnaire was administered to opioid maintenance treatment staff (n=140) and harm reduction staff (n=180). The overall response rate was 84%. The original instrument failed confirmatory analysis. Instead a new two-factor scale was identified and found valid in the new setting.

**Conclusions:**

The failure of the original scale highlights the importance of adapting instruments to current research settings. It also emphasizes the importance of ensuring that concepts within an instrument are equal between the original and target language, time and context. If the described stages in the cross-cultural adaptation process had been omitted, the findings would have been misleading, even if presented with apparent precision. Thus, it is important to consider possible barriers when making a direct comparison between different nations, cultures and times.

## Background

There is much emphasis on using standardized and validated research instruments [1]. One reason for this is the assumption that it enables comparisons of results across different studies both nationally and internationally [1]. Another assumption is that the use of validated instruments increases the certainty with which the instruments accurately reflect what they are supposed to measure [1]. However, a previously validated instrument does not necessarily mean it is valid in another time, culture or context [2-5].

There is no universal agreement on how to adapt an instrument for use in another cultural setting. However, there is agreement that it is inappropriate to simply translate and use a questionnaire in another linguistic context [2,6]. Conversely, studies may have a comprehensive linguistic translation process, but this still does not ensure construct validity and reliability [4,5]. As an example a questionnaire that asks about physical activity and uses cross-country skiing as an example may not be relevant in settings where there is no snow [2]. Moreover, a depression inventory validated in addicted individuals is likely to confuse somatic symptoms of depression with those of intoxication and withdrawal. Additionally, instruments that were validated some time ago may not be valid in the present time due to changes in society that occur continuously [2,3].

In Norway an instrument that measured staff attitudes towards opioid maintenance treatment (OMT) was needed for a study. Staff attitudes towards OMT had never previously been investigated in Norway; consequently there were no instruments available in Norwegian. An Australian-English instrument that measured staff attitudes towards OMT was available. The instrument was developed in NSW, Australia, in 1996 [7]. The instrument had been used in several other studies in Australia [8-11], USA [12-15], Netherlands [16], Germany [17], and Spain [18]. Items tailored towards the country's OMT system were added when the instrument was used outside of Australia [12-18]. However, previous research had not explicitly addressed the cross-cultural adaptation process of the questionnaire.

The cross-cultural adaptation process is important when an instrument is used in a different language, setting and time to reduce the risk of introducing bias into a study [2]. In addition attitudes cannot be measured directly [19]. This means that attitudes are measured indirectly, through some set of items in a questionnaire [19]. In studies where a phenomenon is measured indirectly with questionnaires, comparison of results between cultures and groups may be a challenge. In particular comparison will be difficult if the adaptation process has been flawed. It is therefore important that each item is adapted appropriately.

Thus the aim of this paper is to illustrate the process and required steps involved in the cross-cultural adaptation of a research instrument using the adaptation process of an attitudinal instrument as an example.

## Methods

### A suggested cross-cultural adaption process

Table 1 shows a suggested sequence of the cross-cultural adaptation process. The first stage is to assess if there is the same relationship between the questionnaire and underlying concept in both the original and target setting [2,3]. In addition it is important to assess that items within the instrument are equally relevant and acceptable in the target population as they are in the original population [2]. Both conceptual and item equivalence can be assessed through a literature review [2,3]. Findings from the literature review should be discussed with experts in the field and members of the target population [2,3].

**Table 1:** A suggested cross-cultural adaptation process

Investigation of conceptual and item equivalence	Literature review Discussion with experts in the field and members of target population
Original instrument translated	Translator I: Fluent in target language, good understanding of original language
	
	Translator II: Fluent in target language, good understanding of original language

A synthesized translated version	Translator III: Fluent in target language, good understanding of original language

Back-translations	Back-translator I: Fluent in original language, good understanding of target language
	
	Back-translator II: Fluent in original language, good understanding of target language

A synthesized back-translated version	Back-translator III: Fluent in original language, good understanding of target language

Expert committee	

Instrument pretested	

Revised instrument	

Investigation of operational equivalence	Literature review Discussions with experts in the field and members of target population

Main study	

Exploratory and confirmatory analysis	

Final instrument	

The original instrument should thereafter be translated from the original language into the language of the target population [2-5]. At least two persons should produce the initial translations independently [4-6]. The translators should be fluent in the language of the target population with a good understanding of the original language [2-5]. The translated versions should be synthesized into one version by a third independent translator [4,5]. Thereafter the synthesized version should be back-translated independently by at least two different persons [4-6]. The back-translators should be fluent in the original language with a good understanding of the language in the target population [2-5]. Thereafter the synthesized translated version and the synthesized back-translated version should be reviewed by an expert committee [4,5].

The expert committee should comprise of methodologists, health professionals, language professionals, and the translators (forward and back-translators) [4]. The expert committee assesses if a word or several words reflect the same ideas or subjects in both the original and adapted versions of the questionnaire [2-5]. This assessment ensures that items are translated correctly and are relevant in the new setting [4-6]. If there are uncertainties around the meaning of specific words or items, the developer of the original instrument can be contacted for clarifications [2,4]. It is also suggested to return to the target population and have experts in the field discuss subtleties brought out by the various translation proposals [3]. The instrument should be adjusted accordingly after a consensus is reached [4,5].

Thereafter the instrument should be pretested [4]. Between 30 and 40 respondents are viewed as appropriate in the pretest [3,4]. Respondents are probed for their understanding, acceptability and emotional impact of the items in order to detect confusing or misleading items [3,4]. To ask respondents to rephrase each item is one technique that can identify whether an item is understood or not [3]. Reichenheim (2007) suggests that interviews are conducted until a pre-established percentage of understanding is achieved for all items (e.g. ≥ 90%) [3]. A final semantic adjustment should be made by the research group based on the evidence from the pilot study [3-5].

The operational equivalence of the instrument should be evaluated after the semantic adjustments [2,3]. Operational equivalence means that it is possible to use similar questionnaire format, instructions, mode of administration and measurement methods in the target populations as was used in the original setting [2]. A literature review may give information regarding the use of instruments in the target setting [2]. It is also possible to contact experts in the field and members of the target population to assess if format, instructions, mode and administration and measurement methods are appropriate [2]. Once consensus is reached in regards to operational equivalence, the methods are incorporated into the study [3].

Finally, the instrument should be administered to participants in a formal study. On the basis of the results from this study the psychometric properties of the instrument should be tested using recognized statistical methods [4,5].

### Study instrument

The original study instrument was developed in 1996 [7]. The instrument comprised two attitudinal scales. The 14-item "Abstinence-orientation" scale contained two almost perfectly correlated dimensions: attitudes towards abstinence-oriented policies and support for disciplinary actions if programme rules were broken [7]. Cronbach's alpha for the "Abstinence-orientation" scale was α = 0.89 [7]. The "Disapproval of Drug Use" scale comprised six-items and was characterised by statements such as "modern society is too tolerant towards drug addicts", "marijuana should be legalized", "drug addiction is a vice" and "drug addiction is a menace to society" [7]. The Cronbach's α was 0.75. There was a positive correlation between the "Abstinence-orientation" and "Disapproval of Drug use" scales (r = 0.64) [7].

The responses were answered on a five-point Likert scale ranging from "strongly disagree = 1" to "strongly agree = 5" [7]. A sum score was calculated for each of the two attitudinal scales by dividing the number of completed items by the total score [7].

Additionally the instrument comprised of a 12-item knowledge scale. This scale tested respondents' knowledge of the benefits and risks of methadone treatment [7]. The scale was characterised by statements such as "methadone, when given as a maintenance programme, reduces ("blocks") the effects of heroin" and "methadone maintenance reduces addicts' criminal activities" [7]. The items were scored "1" for correct answer, "0" for "uncertain" and "-1" for incorrect answer [7].

In total there were 32 items in the original instrument. The attitudinal and knowledge items were mixed throughout the instrument [7].

### Main study

The study had a cross-sectional multicenter design. Staff (*n*=140) from the national OMT programme and harm reduction services (*n*=180) in Oslo were invited to participate. The national OMT programme comprised of 14 centres and employed from three to thirty-three staff members. In this study two of the 14 centres were merged because they had a joint staff group at the time of the study. Harm reduction services included street clinics, needle-exchange programs, injecting rooms and housing facilities. The harm reduction services included 12 facilities and employed between six to thirty employees.

Data was collected between August and November of 2007 and was mainly collected through visits by the first author. The researcher was present during the completion of the questionnaires in all except one OMT centre and five harm reduction facilities. In the one OMT centre the researcher gave information during a staff meeting and questionnaires were returned by mail. In one harm reduction facility information about the study was given only to the leader of the facility. Questionnaires from this facility were returned by postal mail. In four harm reduction facilities the researcher gave information during staff meetings and questionnaires were returned by email and mail. Follow-up phone calls were made to ensure that staff returned questionnaires.

The study was approved by the Norwegian Regional Ethics Committee and the Data Inspectorate. Participants received written and oral information about the study. Respondents consented to participate in the study by submitting the questionnaire. The questionnaire was semi-anonymous. This means names were not required, but the name of the facility and other demographic variables made some staff theoretically identifiable. Participants were promised full anonymity. Demographic variables that identify respondents will therefore be deleted upon completion of the project.

### Data analysis

Descriptive statistics were calculated using the statistical software SPSS version 16.0 [20]. Data were assessed using exploratory and confirmatory statistical analysis. Initially the 14 original abstinence-oriented items were tested through confirmatory analysis. The confirmatory analyses were based on the one-factor model developed by Caplehorn, Irwig and Saunders [7]. Subsequently both original and new items were assessed using exploratory factor analysis. The model retrieved through exploratory factor analysis was tested through confirmatory analysis. Exploratory factor analyses were completed in the statistical software SPSS version 16.0 [20]. Confirmatory analyses were completed in the statistical software AMOS graphic version 17.0 [21].

Exploratory factor analyses were completed using principal axis and oblique rotation methods (promax). The correlation matrix and factor loadings were used to decide which items to retain. A scree plot was used to decide the number of factors to retain. Additionally the Cronbach's alpha was assessed.

Confirmatory analyses were completed through structural equation modelling using maximum likelihood analysis. Data were checked for normality both graphically and by assessing univariate and multivariate skewness and kurtosis.

The statistical software AMOS version 17.0 does not handle missing values when modification indices are estimated [21]. Thus a missing value pattern was generated for all items to ensure that values were missing at random. If values were missing at random it would be appropriate to delete missing values listwise for the confirmatory analysis.

The sample data included two different groups (harm reduction staff and OMT staff), thus multigroup analyses were applied. Multigroup analyses were completed stepwise. The steps were 1) the model was tested separately in each group, 2) equal form (unconstrained model) was assessed, 3) equality of factor loadings were tested, 4) equality of structural covariances and 5) equality of measurement errors were assessed. Thereafter if factor loadings and indicator intercepts were invariant, the equality of latent means was assessed [22-24].

There are several goodness-of-fit indices available in maximum likelihood analysis and no agreement on which are best. Goodness-of-fit indices reflect different aspects of the model. It is recommended to report several fit indices to assess how well the hypothetical model fit the sample data [23]. In this study the decision of overall model fit was based upon four fit indices. These indices were the comparative fit index (CFI) [25], the Tucker-Lewis index (TLI) [26] and the root mean square error of approximation (RMSEA) [27]. The average variance extracted (AVE) [28] was also assessed. Aikaike's information criterion (AIC) [29] and the Browne-Cudek criterion (BCC) [30] were assessed to decide best model fit in the multigroup analysis. The standardized residuals and the modification indices were assessed to identify any areas of misfit in the model [31].

CFI is based on a comparison of a hypothesized model and a baseline model [25]. The advantage of this fit index is that it avoids underestimation of fit as it takes sample size into account [23,25]. TLI also addresses the issues of wrongful rejection or acceptance of a model due to sample size [23,26]. Values for both CFI and TLI range from zero to 1.00. Values above 0.90 indicate acceptable fit, whereas values close to 0.95 are indicative of good fit [32].

RMSEA is an attempt to correct for the tendency of the chi-square statistic to reject models with a large *n *or a large number of observed variables [27]. One of the main advantages of RMSEA is that a confidence interval (CI) can be constructed [31]. Values less than 0.05 indicate good fit and values as high as 0.08 represent reasonable errors of approximation in the population [23,24]. Values ranging from 0.08 to 0.10 indicate mediocre fit, and those greater than 0.10 indicate poor fit [23,24].

AVE is a summary indicator of convergence [28]. An AVE of at least 0.50 means that variance explained by the construct is greater than the measurement error [28,31]. AIC and BCC indicate the best trade-off of model fit and parsimony in multigroup analysis [23,29,30]. The model with the smallest estimate indicates the best fit [23].

## Results

### Investigation of conceptual and item equivalence

The research team identified four main concepts within the study instrument. The identified concepts were 1) abstinence-orientated policies in methadone treatment, 2) attitudes towards disciplinary actions if programme rules were broken, 3) attitudes towards drug use in general and 4) knowledge of risks and benefits of methadone treatment. These concepts were identified through reading previous papers that had used the study instrument. In addition each item within the instrument was assessed for potentially irrelevant concepts in the target population.

After a review of the literature, experts and members of the target population were consulted. Based upon the literature review and the general feedback it was decided to omit the six items that made up the "Disapproval of drug use" scale. There were doubts around the relevance of the abstinence-oriented items, but it was decided to retain these items. All other items were retained except for one knowledge item that the scale's original developer suggested be omitted.

### Additional items

It became evident that the instrument lacked certain concepts relevant in the Norwegian setting through discussions and feedback from experts in the addiction field and OMT staff. The main aim of the Norwegian attitudinal study was to identify attitudes that were relevant in the Norwegian setting. Thus it was decided to add items to the instrument. OMT staff and experts in the field were asked to suggest additional items. These suggestions came both via email and in face-to-face meetings. Based upon a subjective judgment of the authors and the feedback from the experts in the field, 12 attitudinal statements were added to the instrument. Examples of these statements are found in italics in table 2. Some items were variants of the original items, while other items introduced new concepts. The additional items were placed after the original items and thus did not alter the instrument's original structure.

**Table 2:** Factor loadings in the "Compliance" and "Accessibility" scale

	OMT staff		Harm reduction staff	
**Compliance items**	**Factor loading**	**Squared multiple correlations†**	**Factor loading**	**Squared multiple correlations†**

OMT patients who ignore repeated warnings to stop using heroin should be gradually withdrawn off methadone	0.630	0.397	0.729	0.532

OMT patients who continue to abuse non-opioid drugs (e.g. benzodiazepines) should have their dose of OMT medication reduced.	0.612	0.374	0.611	0.374

*If repeated warnings of non-prescriptive use of benzodiazepines are ignored, the patient should be discharged from the OMT program*	0.939	0.882	0.948	0.898

*If repeated warnings of use of Cannabis are ignored, the patient should be discharged from treatment (OMT)*	0.845	0.714	0.643	0.413

*The GP should waive the right to prescribe class A and B drugs other than the OMT medication to OMT patients*	0.540	0.292	0.472	0.223

*OMT patients who continue to take drugs and function poorly should be discharged from the OMT program*	0.675	0.455	0.726	0.527

*It is unethical to discharge patients from the OMT program due to continuing drug use and poor functioning*	0.672	0.451	0.690	0.475

**Accessibility items**				

OMT services should be expanded so all heroin addicts who want OMT can receive it	0.635	0.403	0.683	0.466

It is unethical to deny heroin addicts OMT	0.678	0.460	0.743	0.552

*OMT's main aim is to reduce harmful effects of opioids and IV drug use (syringes)*	0.487	0.237	0.310	0.096

*GPs should be able to initiate OMT-medication on their own initiative*	0.288	0.083	0.385	0.148

*Too many LAR-patients are discharged from the OMT program*	0.475	0.226	0.379	0.143

*Young opioid dependents (<20) should not be offered OMT*	0.495	0.245	0.403	0.162

Examples of additional items in italics

† The extent that the variance of the measured variable is explained by the latent factor.

### Forward and back-translations

The original version of the questionnaire was translated from Australian-English to Norwegian by two translators. One of the translators was a health professional and the other translator was not. Both translators were fluent in Norwegian and had good knowledge of English. A third person reviewed the two translated versions and synthesized the two versions into one. This third person was fluent both in Norwegian and English. Both translators agreed on the synthesized version.

Next, the synthesized version was back-translated by two different people. One person was a health professional and one was not. One of the back-translators had English as native language, whereas the other person had lived and studied in the US for many years. The same person, who synthesized the translated versions, reviewed the two back-translations. The two back-translated versions were then synthesized into one. Words that were back-translated differently were highlighted and discussed. When an agreement was reached, the word was added to the synthesized version.

### Review by expert committee

Finally the original instrument, the translated version and the back-translated version were compared by a committee. The committee comprised of PhD students, psychiatrists, medical doctors, one registered nurse and OMT staff. All members of the committee had either full-time or part-time positions at the Norwegian Centre for Addiction Research. All members knew the Norwegian OMT system well. Several members of the committee were fluent in both English and Norwegian, and had completed their degrees in English speaking countries. The instrument was adjusted according to advice from the committee. None of the translators or members of the committee were financially reimbursed.

### Pretest of instrument

A pretest of the instrument (response rate 42/69) was completed among staff working in the addiction field, but not in OMT in May-June 2007. OMT staff were not invited to participate as they were the target population in the main study. Questionnaires were mailed out via email and postal mail. The respondents were asked to complete the questionnaire and comment on words and sentences that were difficult to understand. These comments were written on a paper attached to the questionnaire. There were no criteria for how to reach certain decisions, such as retain or adjust items. Instead this was based solely on the subjective judgement of the researcher and group discussions with experts in the field.

Unclear words and items identified in the pretest were discussed with members of the expert committee and target population. Final adjustments were made based on the subjective judgement of the research team after discussions with members of the expert committee and target population.

### Assessment of operational equivalence

After the final adjustments of the instrument, the instrument format, instructions, mode of administration and measurement methods were assessed by the research group. There was nothing in the format, instructions, mode of administration or measurement methods that was unfamiliar to the Norwegian setting.

### Main study

The overall response rate was 84% (269 out of 320 questionnaires were returned). All staff in the OMT programme (100%), and 129 out of 180 (72%) of harm reduction staff completed the survey.

Reasons given for non-participation in the harm reduction facilities were that staff was not informed and did not have time to complete the survey. One person did not trust the questionnaire would be used only as an anonymous descriptive study and therefore did not complete the questionnaire. Two questionnaires (0.74%) were unusable due to incomplete answers. There were no specific patterns in the missing values. For the confirmatory analysis 21 individuals from harm reduction and 14 individuals from OMT were deleted listwise due to one or more missing attitudinal items.

2/3 of the respondents were women. OMT staff were older than harm reduction staff, with the majority of staff (60%) in the age category 40 to 59 years. All OMT staff had more than three years of tertiary education, whereas 43% of harm reduction staff had less than three years of tertiary education. In addition more OMT staff had worked more than six years in the addiction field compared to harm reduction staff (62% versus 41%).

### Confirmatory analysis of the original abstinence-oriented scale

Data had a normal distribution. The original one-factor abstinence-oriented scale failed confirmatory analysis. This means that the model did not have a good-fit-to the data neither in OMT staff (RMSEA = 0.11 (90% CI 0.09; 0.13), CFI = 0.59, TLI = 0.52, AVE = 0.17) nor in harm reduction staff (RMSEA = 0.12 (90% CI 0.09; 0.14), CFI = 0.58, TLI = 0.51, AVE = 0.19). There was substantial covariance between measurement error 3 and 5 (MI = 19.533) and error 2 and 7 (MI = 31.554) in the OMT group. The model was adjusted accordingly, but the adjusted model did not have an adequate fit to the data. In harm reduction staff, there was a relatively large covariance between error 2 and 7 (MI = 23.807), error 8 and 12 (MI = 11.439) and error 13 and 14 (MI = 11.439). The model was adjusted accordingly, but this model also failed. Table 3 shows the original factor loadings and the factor loadings in the current study. The Cronbach's alpha was α = 0.71 in harm reduction and α = 0.67 in OMT staff. In comparison the Cronbach's alpha was (α = 0.89) when the scale was originally developed.

**Table 3:** Items and factor loadings in the original abstinence-oriented scale

Original abstinence-oriented items		Original factor loadings	OMT staff		Harm reduction staff	
		
			Factor loadings	Squared multiple correlations†	Factor loadings	Squared multiple correlations†
Confrontation is necessary in the treatment of drug addicts		0.53	0.358	0.128	0.556	0.309

*Left to themselves, most methadone patients would stay in maintenance for life*		0.60	0.007	< 0.001	0.013	< 0.001

OMT services should be expanded so all narcotic addicts who want OMT can receive it		0.62	0.238	0.057	0.246	0.061

*Abstinence from all opioids (including methadone) should be the principal goal of maintenance treatment*		0.60	0.516	0.266	0.531	0.282

Methadone maintenance patients who continue to use illicit opiates should have their dose of methadone reduced		0.77	0.699	0.489	0.457	0.209

*No limits should be set on the duration of methadone maintenance*		0.64	0.260	0.067	0.514	0.265

*Methadone should be gradually withdrawn once a maintenance patient has ceased using illicit opiates*		0.62	0.193	0.037	0.558	0.311

It is unethical to deny a narcotic addict OMT		0.54	0.284	0.080	0.334	0.112

OMT patients who ignore repeated warnings to stop using illicit opiates should be gradually withdrawn off methadone		0.76	0.659	0.434	0.594	0.353

Maintenance patients should only be given enough methadone to prevent the onset of withdrawals		0.59	0.115	0.013	0.248	0.062

The clinician should encourage patients to remain in methadone maintenance for at least three to four years		0.51	0.036	0.001	-0.123	0.015

It is unethical to maintain addicts on methadone indefinitely		0.58	0.354	0.126	0.385	0.148

OMT patients who continue to abuse non-opioid drugs (e.g. benzodiazepines) should have their dose of OMT medication reduced.		0.60	0.602	0.363	0.530	0.281

*The clinician's principal role is to prepare methadone maintenance patients for drug-free living*		0.56	0.526	0.277	0.464	0.216

Items in italics omitted from exploratory analysis of a new attitudinal scale

† The extent that the variance of the measured variable is explained by the latent factor.

### Omitted items in the exploratory factor analysis

Five of the 14 original items had to be explained to almost all respondents (items in italics in table 3). Throughout the cross-cultural adaptation process there had been doubts around the relevance of these items in the Norwegian setting. Thus it was decided to omit the five items from the following exploratory factor analysis.

### Assessing original and new items using exploratory factor analysis

The analysis produced a two-factor model including 13 items. The first factor, "Compliance", included seven items (α = 0.89). "Compliance" explained 44% of the variance in the model and the eigenvalue was 5.74. "Compliance" reflected staff attitudes towards sanctions against continuing drug use among OMT patients. The second factor, "accessibility", included six items (α = 0.78) and explained 11% of the variance in the model. Eigenvalue was 1.4. "Accessibility" reflected staff attitudes towards intake criteria in OMT.

### Confirmatory analysis of the model retrieved through exploratory factor analysis

The new attitudinal model had an adequate fit to the data in both groups (Table 4). In the OMT group the model was adjusted to allow covariance between error 1 and 6 (MI = 11.014). The goodness-of-fit indices were improved by this adjustment (Table 4). The model was also improved in the harm reduction group when the model was adjusted to allow covariance between error 6 and 7 (MI = 16.31) and error 11 and 12 (MI = 8.35) (Table 4).

**Table 4:** Confirmatory analysis of the new two-factor attitudinal scale

*Single group analysis*		RMSEA (90% CI)	CFI	TLI	AVE		AIC	BCC
**OMT (n=126)**					**F1**	**F2**		

Unadjusted model		0.072 (0.047; 0.096)	0.928	0.913	0.515	0.276	N/A	N/A

Adjusted*		0.063 (0.034; 0.088)	0.946	0.934	0.509	0.276	N/A	N/A

**Harm reduction (n=108)**								

Unadjusted model		0.079 (0.052; 0.105)	0.905	0.884	0.506	0.267	N/A	N/A

Adjusted model II**		0.049 (0.000; 0.080)	0.964	0.955	0.492	0.261	N/A	N/A

**Measurement Invariance**								

Adjusted model***								

Equal form (Unconstrained model)		0.039 (0.021; 0.053)	0.959	0.947	N/A	N/A	284.528	301.136

Equal factor loading		0.043 (0.028; 0.056)	0.945	0.935	N/A	N/A	288.024	301.587

Equal structural covariances		0.045 (0.031; 0.058)	0.937	0.928	N/A	N/A	292.944	305.677

Equal measurement residuals		0.061 (0.050; 0.072)	0.874	0.868	N/A	N/A	345.446	354.580

Saturated model		N/A	N/A	N/A	N/A	N/A	364.000	414.377

Independent model		0.169 (0.160; 0.178)	N/A	N/A	N/A	N/A	1241.206	N/A

*Allowing covariance between Err1 ↔ Err6, ** Allowing covariance between Err 6 ↔ 7, Err 11 ↔ 12

***Allowing covariance between Err1 ↔ Err6, Err6 ↔ Err7, Err 11 ↔ 12

Multigroup analysis showed that the attitudinal scale differed in all parameters between the two groups (Table 4). The mean "compliance" score for OMT staff was 3.38 (95% 3.23; 3.52) and mean "accessibility" score was 3.51 (95% CI 3.40; 3.62). In harm reduction mean compliance score was 2.54 (95% CI 2.42; 2.67) and mean "accessibility" score was 2.49 (95% CI 2.39; 2.59). It was not possible to assess differences in latent means between the two groups as item intercepts and factors loadings were not equal (Table 4). Factor loadings in the two scales are shown in table 2. There was also a difference in factor covariance between the two groups (Table 4). In OMT staff the covariance between "compliance" score and "accessibility" was 0.32 and in harm reduction staff the covariance was 0.15. Consequently there was also a difference in factor correlations. Factor correlations were 0.71 for OMT staff and 0.37 for harm reduction staff.

Additionally the two groups differed in knowledge scores. OMT staff had a mean knowledge score of 6.19 (95% CI 5.80; 6.58). In comparison harm reduction staff had a mean knowledge score of 3.43 (95% CI 2.92; 3.93).

## Discussion

The failure of the original scale highlights the importance of adapting instruments to current research settings. It also emphasizes the importance of ensuring a single item's and concept's validity in the current language, time and context. The thorough assessment of the Australian attitudinal instrument showed that the concept of abstinence-oriented principles in OMT was not as relevant in the Norwegian setting as it was in Australia in the 1990s. Importantly, if no items had been added at the end of the original instrument, the only findings of this study would have been that the original scale was not valid in Norway. Alternatively, one could have ignored the validity testing intentionally and simply reported findings. However, this would have been misleading and could have given the impression that abstinence-oriented principles in OMT was a current and contentious issue in Norway in 2007.

There are many potential reasons why a cross-culturally adapted scale fails confirmatory analysis. One reason could be a flawed cross-cultural adaption process. This introduces the possibility that the instrument does not measure the same concepts in the original and target settings. The pretest was not conducted according to suggested guidelines [2-5]. Instead respondents were asked to highlight problematic words or items. It would have been more appropriate to ask the respondents to rephrase each item. Furthermore it would have been easier to detect any discrepancies in the instrument if the respondents were interviewed in face-to-face meetings, rather than through mail and email. Additionally decisions for when understanding was achieved for all items were left to the subjective judgement of the researchers. This introduced bias into the cross-cultural adaptation process and may be one of the reasons why the original scale failed.

Alternatively, it may be that the failure of an original scale in a new setting is due to changes in society over time [2]. The original scale was developed in the 1990s. Since then several studies have found that abstinence-oriented treatment in OMT is less effective than long-term maintenance [33,34]. Based upon previous research it may be that the debate has moved on to other issues. Two unpublished studies support this hypothesis. The original scale failed confirmatory analysis in Spain in 2000 and in NSW, Australia in 2003 (*Caplehorn 2007, personal communication*). These findings suggest that the failure of the original abstinence-oriented scale is possibly related to changes over time.

In the cross-cultural adaptation process new items were added to the instrument. It is possible that this altered the structure of the original scale and thus the scale failed confirmatory analysis. However, these items were added at the end of the instrument, after the original items. This means that the structure of the instrument was the same as in the original. The additional items enabled the researchers to find two new attitudinal scales that were valid in the Norwegian setting. Yet the need for additional items suggested that the original instrument was not directly applicable in the new setting.

Another difficulty in the described cross-cultural adaptation process was to locate a second native English-speaking back-translator. This was mainly due to financial constraints. Instead for practical reasons, someone who spoke and wrote English fluently was used as a second back-translator. This illustrates that a thorough cross-cultural adaption process may be difficult to achieve if there are time or financial constraints. Regardless, the subsequent stages after the back-translation presumably detected any discrepancies that might have occurred in the back-translation process. However, it is important to acknowledge that it may have been more appropriate to use someone whose mother tongue was English.

Harm reduction staff were included in this study as a comparison group to OMT staff. It was expected that harm reduction staff would differ greatly from OMT staff. This study confirmed that harm reduction staff differed from OMT staff in age, level of education and experience in the addiction field. They had a lower response rate which was possibly related to the data collection procedure. The researcher was present in all except one OMT centre while respondents completed the questionnaires. In comparison the researcher was only present in seven out of 12 ham reduction facilities during data collection. Potentially it was easier for staff to complete the questionnaire when the researcher was present. Furthermore there were differences between groups in knowledge and attitudinal scores. Importantly multigroup analysis confirmed differences between the groups in all parameters within the new attitudinal scale.

One of these parameters was the correlations between the factors in the new attitudinal scale. There was a higher correlation between the attitudinal factors in the OMT group compared to harm reduction. The differences in correlations suggest that the two factors were more predictive of each other among OMT staff compared to harm reduction staff. If OMT staff believed that no drug use should be tolerated, there was a high possibility they believed the OMT programme should be only for a selected few. Conversely, harm reduction staff who believed drug use should not be tolerated among OMT patients did not necessarily support an OMT programme with limited access. Harm reduction staff were sampled from various institutions and therefore work within different ideologies and traditions. This could explain why there was a lower correlation between the factors among harm reduction staff.

The attitudes of OMT staff are of importance as they are likely to influence treatment practices and, subsequently, treatment outcomes [8,10]. The persistent treatment differences between the Norwegian OMT centres documented through annual assessments [35,36], the high correlations between the two factors within the new attitudinal scale and high mean factor scores support the proposition that attitudes contribute to differences in treatment practices. This needs to be further investigated in a parallel study of staff attitudes and treatment outcomes.

## Conclusions

The failure of the original scale highlights the importance of adapting instruments to current research settings. It also emphasizes the importance of ensuring that concepts within an instrument are equal between the original and target language, time and context. If the described stages in the cross-cultural adaptation process had been omitted, the findings would have been misleading, even if presented with apparent precision. Consequently, it is important to consider possible barriers when making a direct comparison between different nations, cultures and times. There will always be some differences between time-periods and settings, and in many cases, cross-cultural adaptation is recommended even for well established questionnaires.

## Competing interests

The authors declare that they have no competing interests.

## Authors' contributions

LG participated in the conception and design of the study, carried out the data collection, analyzed and interpreted the data and drafted the manuscript. JRMC participated in the analysis and interpretation of the data and revised the manuscript critically for intellectual content. TC participated in the conception and design of the study, participated in the interpretation of the data and revised the manuscript critically for intellectual content. All authors read and approved the final manuscript.

## Pre-publication history

The pre-publication history for this paper can be accessed here:

http://www.biomedcentral.com/1471-2288/10/13/prepub
